# Effect of Caries Infiltrant on Margin Integrity of Composite Fillings Placed Adjacent to Demineralised Primary Enamel

**DOI:** 10.3290/j.ohpd.b2259135

**Published:** 2021-11-05

**Authors:** Rengin Attin, Nathalie Rüedi, Tobias T. Tauböck, Philipp Körner, Daniel Wiedemeier, Thomas Attin

**Affiliations:** a Professor, Clinic for Orthodontics and Pediatric Dentistry University of Zürich, Zürich, Switzerland. Wrote the manuscript.; b Dentist in Private Practice, Niederhelfenschwil, Switzerland. Laboratory work,; c Dentist/Lecturer/Senior Assistant,Clinic for Preventive Dentistry, Periodontology and Cariology, University of Zürich, Zürich, Switzerland. Contributed to study design.; d Dentist/Lecturer, Clinic of Preventive Dentistry, Periodontology and Cariology Center of Dental Medicine, University of Zürich, Zürich, Switzerland. Contributed to study design.; e Statistician, Statistical Services Center of Dental Medicine, University of Zürich, Zürich, Switzerland. Statistical analysis; f Professor, Clinic of Preventive Dentistry, Periodontology and Cariology Center of Dental Medicine, University of Zürich, Zürich, Switzerland. Contributed to study design, proofread the manuscript.

**Keywords:** caries infiltrant, demineralised primary enamel, marginal integrity

## Abstract

**Purpose:**

To investigate the influence of pretreating demineralised enamel with an infiltrant on the margin integrity of Class V like composite restorations on primary teeth bonded with different adhesives.

**Materials and Methods:**

Forty specimens from primary molars were demineralised and circular class-V-like cavities were prepared. The cavities were treated with a universal adhesive (Scotchbond Universal Adhesive, 3M Oral Care), applied either in self-etch (SE) or etch-and-rinse mode (ER) mode. In groups SE-I and ER-I, the demineralised margins were pretreated with a caries infiltrant (Icon, DMG) prior to adhesive application. The cavities were restored with a nanofilled composite material and thermocycled. Marginal integrity was evaluated using SEM, and the percentage of continuous margin was statistically analysed.

**Results:**

Specimens treated with the caries infiltrant followed by the adhesive showed similar marginal continuity as the adhesive alone.

**Conclusions:**

Pretreatment of demineralised primary enamel with a caries infiltrant before applying a universal adhesive does not influence the marginal integrity of composite fillings.

The technique of caries infiltration has been the subject of increasing study in recent years. In order to improve the penetration of the infiltrating resin into the carious lesion, surface conditioning of enamel and composition of the infiltrant have been systematically tested in various in vitro studies.^15,18,23,24^ The infiltration of caries lesions with low viscosity light-curing resins is assumed to be a treatment option for non-cavitated lesions which are not expected to arrest or remineralise. By virtue of a high penetration coefficient, caries infiltrants are able to penetrate the porous body of the lesion almost completely.^15,17^

Due to the low viscosity of the infiltrant, and because the infiltrant does not necessarily require a resin coating, clinical applications on tooth surfaces which are difficult to access, e.g. proximal surfaces, have become possible.^13,26^ It was shown that a caries infiltrant inhibited caries progression under clinical conditions in both primary^5^ and permanent teeth.^22^

In addition, resin infiltrants have also been used to improve the visual appearance of white spot lesions on smooth surfaces and mask them by infiltrating the microporosities.^10,28^

However, extensive active white spot lesions might also reveal cavitated defects, which require additional restorative treatment.^6^ Such lesions might require both infiltration of the demineralised parts as well as restoration of the cavitated areas. Under clinical conditions, the treatment of these lesions could be simplified if the demineralised parts could be infiltrated in the same step as enamel bonding for composite application. In a previous study, it was shown that the adhesion of composite to sound and demineralised permanent enamel was achieved to the same extent by a caries infiltrant and a conventional adhesive.^32^ Moreover, the use of a caries infiltrant before application of a conventional adhesive did not impair bonding to sound and demineralised enamel and might be beneficial as a pretreatment in demineralised enamel.^11,32^

Information is lacking on how far demineralised enamel margins might offer a proper base for tightly sealed restorations. Thus, it might be assumed that under clinical conditions, cavity preparations in primary teeth are often unnecessarily extended into sound adjacent areas in order to prevent secondary caries or marginal leakage.

In permanent teeth, adhesion to demineralised areas could be improved by an infiltrant used before the application of adhesives.^11^ Sufficient adhesion to demineralised enamel of primary teeth would probably lead to less extensive and more prevention-oriented restorations in primary teeth also.

Studies in primary teeth have been performed using adhesives applied to sound dentin and enamel.^12,27,29^ However, little is known about the performance of adhesives applied to demineralised primary enamel.

Therefore, the aim of the present study was to analyse the marginal integrity of composite fillings placed adjacent to demineralised primary enamel, which was pretreated with a universal adhesive or a combination of caries infiltrant and a universal adhesive.

The null hypothesis was that the marginal integrity of restorations placed with the infiltrant in combination with the adhesive on demineralised primary enamel is not statistically significantly different from restorations placed with the adhesive alone.

## Materials and Methods

### Specimen Preparation

Twenty non-carious extracted or exfoliated primary teeth, which were collected for this in vitro test as by-products of regular dental therapy, were anonymised. Extraction of these teeth was neither indicated nor performed by dentists involved in the present study. After cleaning, the teeth were stored in 0.1% thymol solution to avoid dehydration. All parents of patients gave written informed consent that the teeth could be used for research purposes. The teeth were sectioned at the cementoenamel junction and cut in half in the bucco-oral direction, using a water-cooled cutting wheel (Struers; Ballerup, Denmark). The crowns were embedded in self-curing resin (Paladur, Heraeus Kulzer; Hanau, Germany) parallel to the ground. For demineralisation, the specimens were immersed for ten days in an acid buffer. The acidified gel system was made by mixing 0.1 M lactic acid and 0.1 M sodium hydroxide in a proportion that yielded a pH of 4.5, and gelled with 6% w/v hydroxylethyl-cellulose.^4^

After demineralisation, cavity preparations (1.5 mm width and 2.0 mm depth) placed in the middle of demineralised enamel were manually prepared using a spherical-headed diamond bur 1.2 mm in diameter with an average grain size of 80 to 100 µm in a highspeed contra-angled, water-cooled handpiece (Castellini; Bologna, Italy).

### Bonding Procedure

The specimens were randomly assigned to four groups (n = 10 per group). The enamel surface was treated with either an adhesive or a combination of the infiltrant and an adhesive as follows:

1. In group SE (self-etch mode), the universal adhesive (Scotchbond Universal Adhesive, 3M Oral Care) was applied to the enamel and dentin surfaces and gently rubbed in for 20 s with a microbrush, then thinned and evaporated with a mild air stream (5 s), and light cured for 20 s (Bluephase G2, Ivoclar Vivadent; Schaan, Liechtenstein).
2. In group ER (etch-and-rinse mode), the enamel and dentin surfaces were etched with 35% phosphoric acid (UltraEtch, Ultradent; South Jordan, UT, USA) for 15 s and then rinsed with water spray for 10 s. Air drying of the surface was followed by application of the adhesive (Scotchbond Universal Adhesive, 3M Oral Care) for 20 s, air thinning for 5 s, and light curing for 20 s.
3. In group SE-I (self-etch mode in combination with the infiltrant), the enamel surface was etched with 15% hydrochloric acid gel (Icon Etch, DMG) for 120 s and then rinsed with water for 30 s. Then, the surface was dried with ethanol (Icon Dry, DMG) applied for 30 s. Thereafter, the low-viscosity infiltrant resin (Icon Infiltrant, DMG) was applied to the surface for 180 s and light cured for 40 s. After light curing, the infiltrant was applied again for 60 s and light cured for 40 s. Then, the universal adhesive was applied as described in group 1.
4. Samples of group ER-I (etch-and-rinse mode in combination with the infiltrant) were first treated with infiltrant as described in group 3. Then, the universal adhesive was applied as described in group 2.

The composition of the universal adhesive and the caries infiltrant system according to the respective manufacturers is listed in Table 1.

**Table 1:** Composition of the caries infiltrant system (Icon) and the universal adhesive (Scotchbond Universal Adhesive)

Product	Composition*	Manufacturer
Icon	Icon-etch: 15% hydrochloric acid, water, silicic acid, tenside, pigments Icon-dry: 99% ethanol Icon-infiltrant: TEG-DMA-based resin matrix, initiators, additives	DMG; Hamburg, Germany
Scotchbond Universal Adhesive	MDP phosphate monomer dimethyacrylate resins HEMA, Vitrebond copolymer, filler, ethanol, water, initiators, silane	3M Oral Care; St Paul, MN, USA

*Manufacturers’ information. TEG-DMA: triethylene glycol dimethacrylate; MDP: methacryloyloxydecyl dihydrogen phosphate; HEMA: 2-hydroxyethyl methacrylate.

The pretreated cavities were filled with a nano-filled composite (Filtek Supreme XTE, 3M Oral Care; St Paul, MN, USA) in one increment and light cured for 20 s. Bonding procedures were carried out by the same operator throughout the experiment.

The restorations were polished with wet silicon carbide abrasive papers, brownies, greenies, and polishing disks (Shofu Brownie FG Mini Points, Shofu Greenie FG Mini Points, Sof-Lex Polierscheiben XT 2382SF, 2382F, 2382M; Kyoto, Japan). The specimens were thermocycled (Willytec; Gräfenling, Germany) 5000 times in water between 5°C and 55°C (dwell time in each temperature bath: 20 s) prior to marginal integrity testing.

### Scanning Electron Microscopy

For replica preparation, each specimen was impressed in polyvinylsiloxane impression material (President Light Body, Coltene; Altstätten, Switzerland), and positive replicas were poured with epoxy resin (Epoxyharz L, R&G Faserverbundwerkstoffe; Waldenbuch, Germany).

The replicas were gold coated and inspected using SEM (ERA-8800FE, Elionix; Tokyo, Japan).

**Fig 1:**
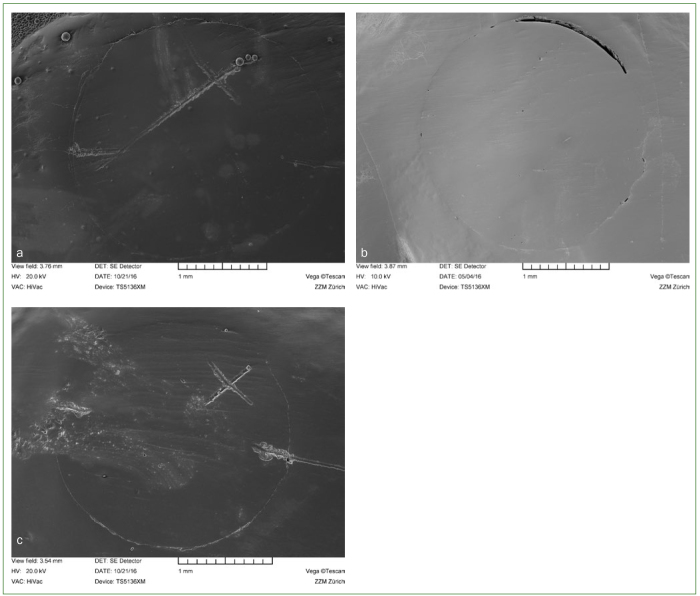
Criteria for marginal analysis: SEM images for continuous (a), non-continuous (b), and non-assessable margins (c).

Margins were classified as ‘continuous’, ‘non-continuous’ or ‘not assessable’ (Fig 1). The marginal integrity of the restorations was expressed as percentage of ‘continuous margin’ relative to the total assessable margin.

### Statistical Analysis

Descriptive statistics for percentage of continuous margin (mean ± standard deviation, 95% confidence interval) were computed and statistically analysed using SPSS software (SPSS; Chicago, IL, USA). The assumption of normal distribution of data was checked (Kolmogorov-Smirnov test), and one-way ANOVA was subsequently performed. The level of significance was set at 5%.

## Results

**Fig 2:**
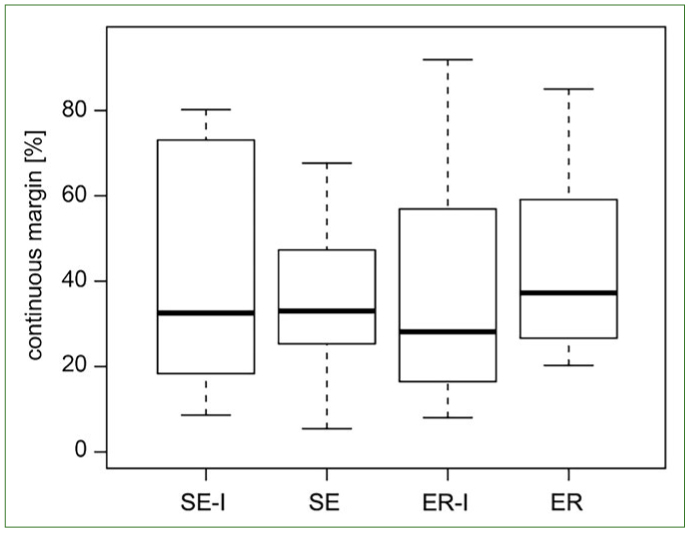
Box plots of continuous margins [%] in the different experimental groups (SE: self-etch mode; ER: etch-and-rinse mode; I: infiltrant) in demineralised primary enamel.

Marginal integrity of primary enamel treated with a universal adhesive or a combination of the caries infiltrant and the adhesive is presented in Fig 2.

No statistically significant difference was detected between the groups. Specimens treated with the caries infiltrant followed by the adhesive showed marginal continuity similar to those receiving the adhesive alone.

## Discussion

This study demonstrated that the marginal continuity of nanofilled composite restorations placed adjacent to demineralised primary enamel using an infiltrant system in combination with a universal adhesive is comparable to the marginal integrity when using the universal adhesive alone. Thus, the null hypothesis was not rejected.

Artificial enamel lesions were created following previous studies investigating resin infiltration in vitro and were shown to exhibit the typical histological structure of enamel caries.^1,14,16,19^

Specimens were treated with the caries infiltrant system or the universal adhesive, following manufacturers’ recommendations. Thus, etching was performed using 15% hydrochloric acid or 35% phosphoric acid, respectively. A previous study demonstrated that the surface layer of caries lesions can be eroded almost completely by 15% hydrochloric acid, but not by phosphoric acid, without destroying the underlying surface.^1^ This feature allows better resin penetration into the bulk of the carious lesion.^5,21^ Thus, not only the resin itself, but also the kind of etching might have influenced the marginal continuity.

Based on the current trends toward ease-of-use and faster clinical application steps, Scotchbond Universal Adhesive was chosen as a representative of a universal adhesive. Aiming to eliminate complications and provide a single product for all situations, universal adhesives that can be applied in both self-etch and etch-and-rinse mode^7,25^ have been recently introduced. Previous studies in dentin showed that the addition of an etching step did not significantly affect the adhesive performance of a universal adhesive,^29^ i.e. Scotchbond Universal Adhesive, when compared to the self-etch application mode.^31^ However, this topic is somewhat controversial, since other studies observed the opposite results.^3,20^

As shown previously, high amounts of TEG-DMA and ethanol in experimental resin infiltrants enhance penetration ability by decreasing their viscosity and contact angle to enamel.^15,24^ On the other hand, increasing amounts of TEG-DMA often cause inhomogeneities, probably as a result of polymerization shrinkage and polymerization stress of the resin,^24^ which might increase the risk of leakage and thus might affect marginal continuity. However, the present findings indicate that the adhesion of the TEG-DMA-based resin infiltrant is not affected such that marginal continuity is statistically significantly reduced.

Although universal adhesives might penetrate demineralised enamel to a lesser extent than infiltrants, penetration might be more homogenous compared to infiltration. As a result, the adhesion of a nano-filled composite might be achieved to a similar extent by the more complete, but inhomogeneous infiltration (caries infiltrant system) and the superficial, but more homogenous layer (adhesive).

As shown in a previous study,^32^ the adhesive performance of permanent teeth was slightly increased when the infiltrant and adhesive were combined. This was not the case for primary teeth in the present study. Under this premise, it must be borne in mind that the additional pre-treatment step using a caries infiltrant would substantially increase the total treatment costs. Thus, the procedure of using an infiltrant in addition to an adhesive might be uneconomical and would tend towards over-treatment.

Since primary and permanent teeth present differences in their chemical composition, physical structure and micromorphology, these characteristics may interfere with the adhesive performance and explain these differences.^2,8,9,30^

Furthermore, the limited etching time of 30 s as used in the tested primary teeth was set respecting the often-compromised clinical conditions in paediatric dentistry and according to the etching time recommended for permanent teeth. It might be speculated that longer etching times or bevelling of the cavity margins might have led to better results.

## Conclusion

The use of a caries infiltrant before application of a universal adhesive does not affect bonding to demineralised primary enamel. Further studies are needed to examine whether the risk of secondary caries at restoration margins could be lowered by treating demineralised enamel with the infiltrant prior to the application of an adhesive.
